# Fronto-Parietal Brain Metabolites Following Traumatic Brain Injury—A Proton MR Spectroscopy Study

**DOI:** 10.3390/brainsci16101088

**Published:** 2026-10-09

**Authors:** Ping-Hong Yeh, Chen-Haur Yeh, Terrence R. Oakes, John Ollinger, Gerard Riedy

**Affiliations:** Department of Research, National Intrepid Center of Excellence, Walter Reed National Military Medical Center, Bethesda, MD 20889, USA

**Keywords:** proton magnetic resonance spectroscopy imaging (^1^H-MRSI), traumatic brain injury (TBI), blast injury, choline, N-acetylaspartate, post-traumatic stress symptoms

## Abstract

**Background:** Conventional structural CT and MRI may be normal after mild traumatic brain injury (mTBI), while magnetic resonance spectroscopic imaging (^1^H-MRSI) can provide regional measures of brain metabolites. We examined supracallosal fronto-parietal metabolite ratios in active-duty service members with military-related TBI and non-TBI military controls, including comparisons by injury mechanism and exploratory clinical associations. **Methods**: The study used a retrospective cross-sectional cohort. Participants underwent 3T 2D multivoxel ^1^H-MRSI covering a 6 × 8 × 1 cm^3^ supracallosal fronto-parietal PRESS/CSI volume of interest. Regional N-acetylaspartate (NAA), choline-containing compounds (Cho), and creatine/phosphocreatine (Cr) measures were processed with SIVIC and summarized as NAA/Cr and Cho/Cr ratios. Linear mixed-effects models were adjusted for age and sex, with Bonferroni correction for regional group comparisons. Male-only analyses were performed as a sensitivity analysis. Associations with clinical and neurocognitive measures were exploratory. **Results**: The final analytic cohort reported in the manuscript comprised 741 service members with TBI (34.3 ± 8.1 years old; M/F = 711/30; mean 327.4 ± 226.8 days post-injury) and 75 non-TBI controls (M/F = 53/22; mean age: 33.1 ± 10.0 years). TBI participants showed lower Cho/Cr area ratios in multiple fronto-parietal regions, including right precuneus (estimate = −0.0781, SE = 0.0176, t = −4.43, pBonf < 0.0001, Cohen’s d = −0.36, 95% CI [−0.1127, −0.0435]), left superior frontal gyrus (estimate = −0.0715, SE = 0.0163, t = −4.38, pBonf < 0.0001, Cohen’s d = −0.36, 95% CI [−0.1035, −0.0394], and bilateral fronto-parietal WM (right: estimate = −0.0741, SE = 0.0169, t = −4.39, pBonf < 0.0001, Cohen’s d = −0.35, 95% CI [−0.1072, −0.0410]; left: estimate = −0.0656, SE = 0.0161, t = −4.08, Cohen’s d = −0.33, pBonf = 0.0011, 95% CI [−0.0971, −0.0340]). Injury mechanism stratification revealed a widespread reduction in Cho/Cr area in individuals with blast-related TBI relative to unexposed non-TBI controls over the left superior frontal gyrus (estimate = −0.0941, SE = 0.0176, t = −5.35, pBonf < 0.0001, Cohen’s d = −0.44, 95% CI [−0.1287, −0.0596]), right precuneus (estimate = −0.0900, SE = 0.0193, t = −4.66, Cohen’s d = −0.38, pBonf = 0.0010, 95% CI [−0.1279, −0.0521]), and right fronto-parietal WM (estimate = −0.0895, SE = 0.0182, t = −4.91, Cohen’s d = −0.39, pBonf < 0.0001, 95% CI [−0.1253, −0.0537]). Twelve regional differences remained significant in the primary analysis, and the principal pattern was substantially preserved in the male-only sensitivity analysis. Blast-related and multiple-blast comparisons also showed regional Cho/Cr differences; individuals with multiple blast exposures mainly drove Cho/Cr reduction. Exploratory clinical analyses found inverse associations between Cho/Cr and Post-Traumatic Stress Checklist–Civilian version (PCL-C) and Neurobehavioral Symptom Inventory (NSI) scores, and positive associations between selected NAA/Cr measures and cognitive performance. Specifically, higher NAA/Cr area over the left caudal anterior cingulate correlates positively with verbal fluency performance (r = 0.358, *p* < 0.0001). Higher NAA/Cr peak heights in the left caudal middle frontal cortex correlated with better response inhibition accuracy (r = 0.278, *p* = 0.0003). Concurrently, lower Cho/Cr values over default mode network hubs—specifically the left caudal anterior cingulate gyrus (estimate = −0.00144, t = −4.02, uncorrected *p* < 0.0001, 95% CI [−0.00215, −0.00074]), left posterior cingulate gyrus (estimate = −0.00115, t = −3.76, uncorrected *p* = 0.00019, 95% CI [−0.00175, −0.00055], and the left precuneus gyrus (estimate = −0.00120, t = −3.62, *p* = 0.00033, 95% CI [−0.00186, −0.00055])—associated significantly with greater self-reported post-traumatic stress severity on the PCL-C. In addition, Cho/Cr in the left fronto-parietal WM was inversely associated with PCL-C (estimate = −0.00115, t = −3.87, *p* = 0.00012, 95% CI [−0.00174, −0.00057]) and NSI (estimate = −0.00117, SE = 0.00030, t = −3.92, *p* < 0.0001, 95% CI [−0.00176, −0.00058]). Injury chronicity did not correlate with metabolic alterations. **Conclusions**: ^1^H-MRSI identified regional metabolite-ratio differences in this military TBI cohort. The findings support further investigation of regional Cho/Cr and NAA/Cr measures as correlates of clinical heterogeneity after TBI. The widespread depression of Cho/Cr suggests persistent membrane depletion and/or chronic axonal degeneration, particularly in TBI individuals with multiple blast exposures. Although the cross-sectional design does not establish causality, diagnostic sensitivity, biomechanical strain patterns, or longitudinal recovery, the results imply the utility of MRSI as a robust translational tool for phenotyping chronic military brain injury and objectively monitoring long-term neural recovery.

## 1. Introduction

Traumatic brain injury (TBI) is common among military service members and may result from blast exposure, blunt impact, falls, motor vehicle collisions, or combinations of mechanisms [1,2]. Although many individuals with mild TBI (mTBI) recover, some report persistent cognitive, neurobehavioral, and psychological symptoms [3]. Conventional computed tomography (CT) and magnetic resonance imaging (MRI) can be normal after mTBI [4], and an abnormal structural study is not required for the diagnosis; when trauma-related abnormalities are present, the injury may be classified as complicated mTBI [5,6,7,8]. These features motivate investigation of complementary imaging measures [9].

Proton magnetic resonance spectroscopic imaging (^1^H-MRSI) measures regional concentrations or signal ratios of brain metabolites and therefore provides biochemical information that differs from structural MRI offers a powerful tool to address this limitation by providing a window into the microstructural, metabolic, and neurochemical integrity of brain tissue well before cellular degradation manifests as macrostructural volume loss [10,11,12]. N-acetylaspartate (NAA) is localized almost exclusively within neurons and axons, serving as a well-established surrogate marker for neuronal viability and axonal structural integrity [13]. Choline (Cho) compounds are related to membrane phospholipid metabolism and turnover, although the Cho signal is not specific to a single cellular or inflammatory process [14,15,16]. Creatine (Cr)/phosphocreatine represents a relatively stable index of total cellular energetics and basic metabolic homeostasis, routinely utilized as an internal reference standard to derive normalized metabolic ratios (NAA/Cr, NAA/Cho, and Cho/Cr), but it is not necessarily invariant following TBI [11,15,17].

Military TBI may involve multiple concurrent mechanisms, making direct separation of blast and non-blast effects difficult. The supracallosal fronto-parietal region is of interest because it contains cortical and white matter (WM) structures involved in cognitive and behavioral functions, vulnerable to the biomechanical forces during blunt impacts and shockwave compression-rarefaction cycles during explosive blast exposures [18]. Prior MRS studies have reported heterogeneous changes in NAA, Cho, and Cr after TBI, with differences related to injury severity, brain region, and time from injury [19,20]. Despite extensive pre-clinical modeling, an ongoing debate persists regarding whether blast-induced TBI mechanisms introduce a distinct biochemical phenotype compared to traditional blunt or non-blast impact mechanics, or if they primarily reflect varying degrees of severity along a single pathophysiological continuum.

The objective of the present study was to leverage a massive cohort of over 700 active-duty military service members with chronic TBI to systematically map fronto-parietal metabolic profiles using multi-voxel 2D ^1^H-MRSI. We aimed to: (1) evaluate regional alterations in metabolic ratios between individuals with chronic TBI and non-TBI military controls; (2) elucidate whether blast-induced injury mechanisms present unique localized metabolic signatures relative to blunt/non-blast impact trauma; and (3) establish the translational relevance of these neurochemical metrics by mapping their direct correlations with objective neurocognitive performance and self-reported psychological symptom severities. We hypothesized that chronic military TBI would be characterized by diffuse reductions in axonal and membrane integrity markers across fronto-parietal subregions [21] that blast-exposed individuals would display distinct regional vulnerabilities, and that these metabolic deficits would significantly correlate with objective executive slow-down and elevated post-traumatic stress symptoms.

## 2. Materials and Methods

This retrospective cross-sectional study was prepared in accordance with STROBE guidance. Ethical approval was obtained from the institutional review board at the Walter Reed National Military Medical Center (WRNMMC) in Bethesda, Maryland, and the HIPAA-compliant Neuroimaging Core project. All subjects enrolled in the study voluntarily and provided written informed consent prior to the initiation of any experimental procedures.

Participant Recruitment and Demographics

**Figure 1:**
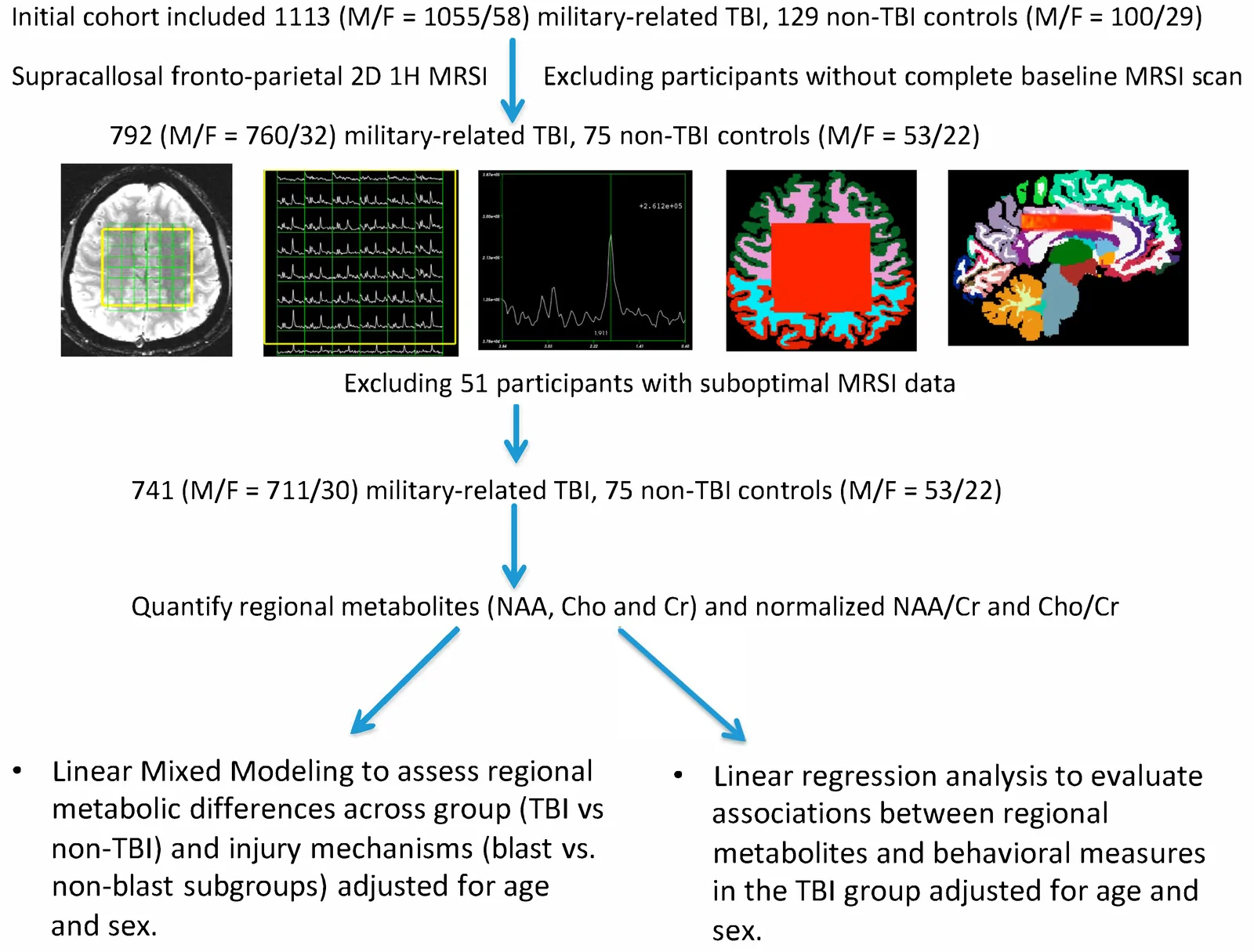
Participant Enrollment and Study Flowchart. Methodologic overview of the study design and multi-voxel ^1^H-MRSI region of interest placement, tissue segmentation, parcellation workflow, as well as regional brain metabolite analysis and brain-behavioral analysis. Representative axial structural T1-weighted magnetic resonance imaging slices depicting the systematic spatial localization and planning of the 2D chemical shift imaging point-resolved spectroscopy region of interest (ROI) across the supracallosal fronto-parietal region. A representative processed single-voxel spectrum extracted from the fronto-parietal matrix demonstrating well-resolved resonance peaks for N-acetylaspartate (NAA, 2.02 ppm), Choline (Cho, 3.22 ppm), and Creatine (Cr, 3.02 ppm) following 6 Hz Lorentzian apodization and phase correction. Three-dimensional cortical surface reconstruction and automated anatomical parcellation were executed via the FreeSurfer neuroimaging suite, highlighting the structural gray matter and white matter within the region of interest (red box delineating the supracallosal fronto-parietal ROI) used to sample localized metabolite grids.

Participants were active-duty service members aged 18–60 years. TBI participants were recruited through the National Intrepid Center of Excellence (NICoE) Intensive Outpatient Program, who had consented to participate in the Neuroimaging Core Study of the NICoE; and non-TBI controls came from the local community who were Defense Enrollment Eligibility Reporting System (DEERs) eligible and consented to the Neuroimaging Core Study (2010–2016) (Figure 1). Eligibility and exclusion criteria included ability to provide consent, absence of disqualifying neurological or psychiatric conditions unrelated to the injury or deployment, absence of MRI-incompatible metal implants or shrapnel, and conversational English proficiency. TBI history was obtained through a clinical interview addressing loss of consciousness (LOC), post-traumatic amnesia (PTA), alteration of consciousness (AOC), injury circumstances, and military exposure history. mTBI classification used the Glasgow Coma Scale (GCS), PTA, LOC, and AOC criteria; trauma-related CT/MRI abnormalities were used to distinguish complicated from uncomplicated mTBI. Final classification was established by clinical case conference. Inter-rater reliability was not formally assessed.

Neuropsychological and Clinical Assessments

Clinical measures included the Neurobehavioral Symptom Inventory (NSI), Post-Traumatic Stress Checklist–Civilian version (PCL-C), and selected neuropsychological measures from Wechsler Adult Intelligence Scale [WAIS-IV] and Delis-Kaplan Executive Function System [D-KEFS] administered according to clinical judgment. The available measures included cognitive, language, executive, verbal-fluency, inhibitory-control, and processing-speed domains. The neurobehavioral and psychological assessments were used to characterize symptom burden rather than to establish psychiatric diagnoses from the MRSI analysis. The PCL-C was treated as a measure of post-traumatic stress symptom severity, while the NSI captured broader post-concussive/neurobehavioral symptoms. Cognitive testing was not necessarily available for every participant because selected measures were administered according to clinical judgment.

Magnetic Resonance Data Acquisition

MRI was acquired on a 3T GE MR750 scanner with a 32-channel head coil. T1-weighted BRAVO was acquired with TI 450 ms, TR 6.7 ms and TE 2.5 ms. ^1^H-MRSI was acquired using a 2D multivoxel chemical-shift imaging (CSI) sequence with PRESS localization, and a 6 × 8 × 1 cm^3^ volume was positioned over the supracallosal fronto-parietal region using sagittal and coronal structural images. Importantly, the 6 × 8 × 1 cm^3^ volume of interest was over the supracallosal fronto-parietal region. These dimensions describe the overall PRESS/CSI volume of interest, not an individual spectroscopic voxel (Figure 1).

MRSI Reconstruction and Metabolite Quantification

Raw MRSI data were processed with Spectroscopic Imaging VIsualization and Computing (SIVIC, https://github.com/SIVICLab/sivic) [20]. A 6 Hz Lorentzian line-broadening filter was applied, followed by 2D Fourier reconstruction, phase correction, and coil combination. NAA, Cho, and Cr were quantified using peak height and integrated peak area. NAA/Cr and Cho/Cr ratios were calculated as normalized measures rather than absolute concentrations.

SNR-Based Spectral Quality Control

Spectral quality control used the SIVIC svk_noise procedure. Participant datasets with noise values > 150,000 were excluded. Metabolite-specific SNR thresholds were applied to integrated-area measures (NAA 10, Cho 5.58, Cr 5.8) and peak-height measures (NAA 2.32, Cho 1.60, Cr 1.67). The analysis used the central 6 × 8 voxel grid, separated by hemisphere, and regional means were calculated. Values coded as 0 or 999,999 were treated as missing; regional measures were retained when more than 50% of participants had available data. FreeSurfer [22] was used for T1-based anatomical parcellation, and anatomical masks were registered to the MRSI grid to define regional gray- and white-matter measures. Standardized retrospective FWHM/linewidth and motion thresholds were not available in the final analysis dataset and therefore were not imposed. The SNR procedure was applied before regional averaging. The participant-level noise threshold and metabolite-specific thresholds were used to limit analyses to spectra with adequate signal. However, retrospective quantitative linewidth/FWHM and motion thresholds were not retained as standardized variables in the final dataset.

For regional quantification, SIVIC-generated metabolite maps were thresholded using the prespecified SNR criteria and restricted to the central MRSI grid. Left- and right-hemisphere regional measures were then summarized within anatomical masks derived from the structural T1 image. Both integrated-area and peak-height measures were retained. To reduce the influence of intersubject and acquisition-related scaling differences, metabolite measures were additionally expressed as ratios to creatine (NAA/Cr and Cho/Cr). These ratios should be interpreted as internally normalized metabolite measures rather than as absolute metabolite concentrations.

Statistical Analysis

Statistical analyses used separate linear mixed-effects models (PROC MIXED, SAS Institute, Cary, NC, USA, v3.82) for each metabolite measure and region. Group was the primary fixed effect, with age and sex as fixed covariates; participant ID was the subject variable, and compound symmetry was used for within-participant covariance. Least-squares means, pairwise comparisons, and 95% confidence intervals were obtained, with Bonferroni adjustment across pairwise comparisons. Group-by-region and group-by-hemisphere interactions were not included and were not formally tested. Each metabolite measure and anatomical region was analyzed in a separate model, rather than fitting a single model containing region-by-group interactions. Consequently, the significant results identify regions in which the estimated group contrast differed from zero after adjustment; they do not demonstrate that one region has a statistically larger effect than another.

Because of the marked sex imbalance, a male-only sensitivity analysis used the same model with age as a covariate. The primary analysis pooled the TBI cohort for comparison with the non-TBI control group. Mechanism-specific analyses then separated blast-related and non-blast TBI and considered non-TBI controls with and without reported remote blast exposure. These subgroup comparisons were not interpreted as randomized or independent tests of injury biomechanics. The male-only analysis was included specifically to examine sensitivity to the marked difference in sex distribution between groups.

Clinical correlations in TBI participants were evaluated with age- and sex-adjusted Pearson partial correlations/linear regression (REG procedures, SAS Institute, Cary, NC, USA, v3.82) and are treated as exploratory. Due to high dimensionality, results were evaluated against a Bonferroni-corrected *p* < 0.05 and a stringent nominal threshold of *p* < 0.001.

## 3. Results

Demographic and Injury Profile

In this retrospective cross-sectional study, the initial cohort comprised 1113 (M/F = 1055/58) active-duty military service members with TBI and 129 non-TBI controls (M/F = 100/29). Among them, 792 (M/F = 760/32) TBI participants and 75 non-TBI controls (M/F = 53/22) had completed MRSI baseline scans. Furthermore, 51 TBI participants with suboptimal MRSI data and 4 TBI participants with missing injury history were excluded. The final cohort comprised 741 active-duty service members with a confirmed medical history and clinical diagnosis of traumatic brain injury (TBI). Within this TBI group, the demographic distribution was predominantly male (M/F = 711/30) with a mean cohort age of 34.3 ± 8.1 years. The average time elapsed between the index injury and the neuroimaging evaluation was 327.4 ± 226.8 days, establishing a chronic post-injury profile. The demographics and baseline clinical phenotypes of both cohorts are detailed in Table 1. Subjects within the TBI cohort were further stratified by injury biomechanics into two distinct mechanical subgroups. Stratification of injury mechanism yielded a blast-induced TBI subgroup of 535 (M/F = 523/12) participants who sustained an injury primarily attributed to an explosive blast wave, and 206 (M/F = 188/18) participants whose injuries resulted from impact, falls, or motor vehicle collisions without blast exposure. For comparative analysis, the final non-TBI control cohort consisting of 75 active-duty service members or matched military controls was recruited (M/F = 53/22; mean age: 33.1 ± 10.0 years). Notably, six male participants within this non-TBI comparison group had a history of remote operational blast exposure but did not meet the diagnostic threshold for a clinical diagnosis of TBI. The cohort was predominantly male, with no significant difference in mean age and sex distribution between TBI and the control groups.

Sex Distribution and Sensitivity Analysis

The final MRSI analytic cohort included 741 participants with military-related mTBI (711 male and 30 female) and 75 non-TBI controls (53 male and 22 female). Thus, females represented 4.0% of the mTBI group and 29.3% of the non-TBI control group. The sex distribution differed significantly between the groups (Fisher’s exact test, *p* < 0.001).

Regional Metabolic Alterations: TBI vs. Controls

**Figure 2:**
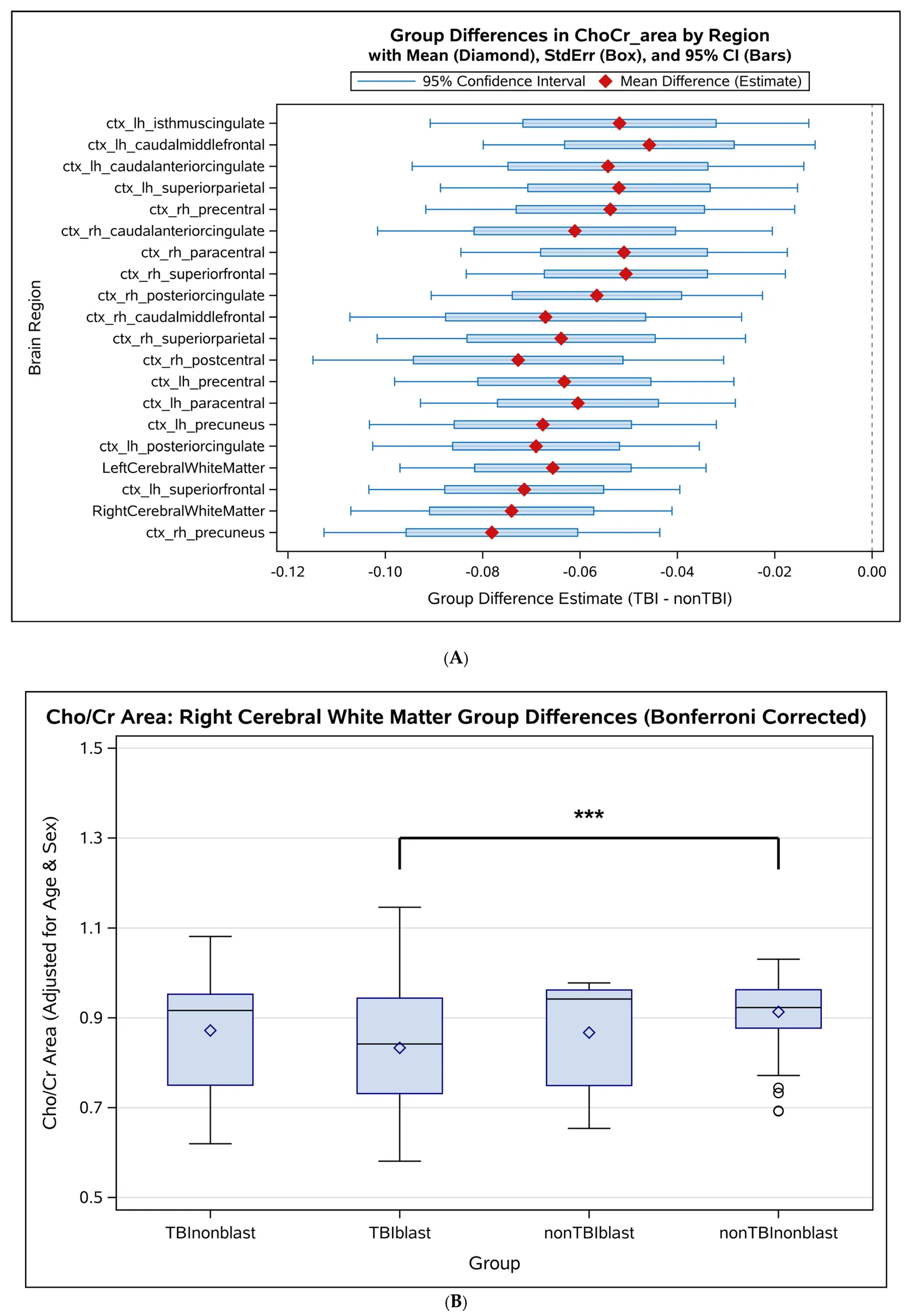

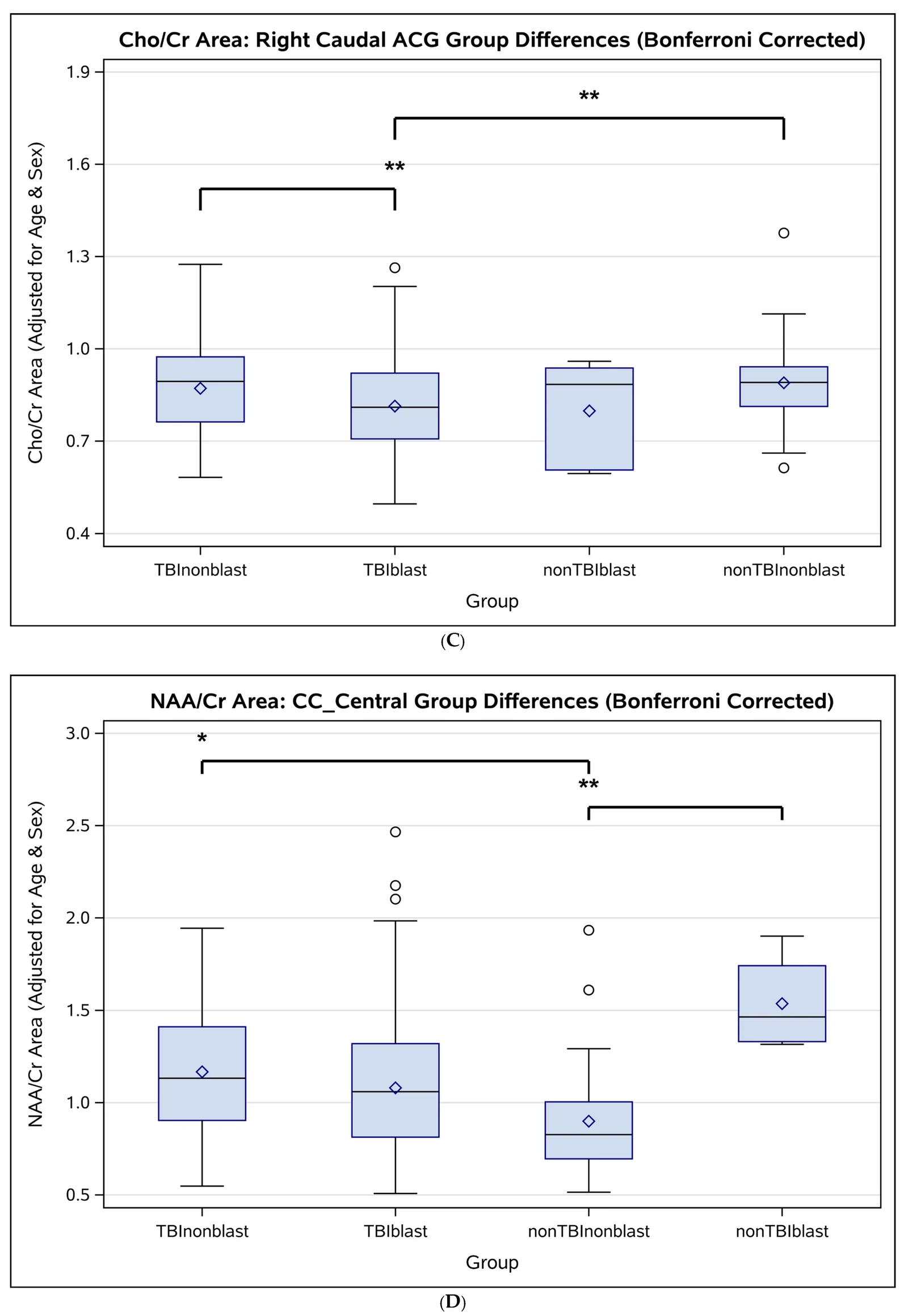
Graphical Representation of Group Differences in Regional Brain Metabolites. Mean difference (diamond), standard error bounds (box), and 95% confidence intervals (whiskers) for brain metabolites across distinct brain regions. (**A**) Boxplots detailing localized group differences (Mean ± Standard Error) in Cho/Cr area ratios: the TBI group had a lower Cho/Cr area ratio than controls over the bilateral fronto-parietal white matter, as well as other subregions (see Table 2) ctx: cortex. (**B**) Blast-induced TBI subgroup exhibits a significantly lower Cho/Cr area ratio compared to the non-blast impact subgroup and non-TBI non-blast controls over the right fronto-parietal white matter. (**C**) Blast-associated TBI showed greater reductions in Cho/Cr area within the right caudal anterior cingulate (ACG) than non-blast TBI (Estimate = −0.0597, t = −3.66, pBonf = 0.0060, d = −0.30) and non-TBI control without blast exposures (Estimate = −0.0865, t = −3.90, pBonf = 0.0023, d = −0.32). (**D**) Non-TBI with previous blast exposure and non-blast TBI participants exhibited significant increases in NAA/Cr area in the central corpus callosum than non-TBI control without previous blast exposures (Estimate = 0.6506, t = 3.58, pBonf = 0.0097, d = 0.38; Estimate = 0.2781, t = 3.37, pBonf = 0.0208, d = 0.36, respectively). * indicates *p* < 0.05, ** indicates *p* < 0.01, *** indicates *p* < 0.001, Bonferroni-corrected.

Linear mixed models, adjusting for age and gender as fixed covariates, revealed extensive metabolic differences between the total TBI cohort and non-TBI controls within the supracallosal fronto-parietal region. Table 2 summarizes the regional fronto-parietal metabolite ratios across cohorts. Figure 2A shows the bar charts detailing localized group differences (Mean ± Standard Error) in Cho/Cr area ratios; the TBI group had a lower Cho/Cr area ratio than controls over the bilateral fronto-parietal white matter, as well as other sub-regions (see Table 2).

No significant differences were observed in total regional Cr levels, validating its utility as an internal reference marker across the cohorts.

Male-Only Sensitivity Analysis (Supplementary Materials)

In the sensitivity analysis restricted to male participants, 12 regional Cho/Cr area differences remained significant after Bonferroni correction. Ten of the 12 regions overlapped with those identified in the primary analysis, including the right precuneus, bilateral cerebral white matter, left superior frontal cortex, left posterior cingulate, left precuneus, left paracentral and precentral regions, right postcentral cortex, and right posterior cingulate. The right superior parietal and right caudal middle frontal findings from the primary analysis were no longer significant after Bonferroni correction in the male-only analysis. Results of sensitivity analyses are listed in Tables S1–S7. Conversely, bilateral caudal anterior cingulate differences reached Bonferroni-corrected significance in the male-only analysis. Importantly, all significant group differences in the male-only analysis were in the same direction as the primary findings, with lower Cho/Cr in the mTBI group (Table S1). These results indicate that the principal pattern of reduced regional Cho/Cr in mTBI was substantially preserved after restricting the analysis to male participants.

Regional MRS Metabolite Alterations Across Blast and TBI Profiles

In the primary analysis including both male and female participants and adjusting for age and sex, Bonferroni-corrected analyses identified 12 significant regional differences (Bonferroni-adjusted *p* < 0.05). Significant differences were observed in the right precuneus, bilateral cerebral white matter, left superior frontal cortex, bilateral precuneus, bilateral posterior cingulate regions, left paracentral and precentral regions, right postcentral cortex, right superior parietal cortex, and right caudal middle frontal cortex. The adjusted group differences ranged from −0.0781 to −0.0506, with Bonferroni-adjusted *p* values ranging from 0.000248 to 0.026806 (pBonf < 0.05, Table 2).

Cortical and White Matter Choline Deficits in Blast TBI

The most prominent pattern was a widespread reduction in choline-to-creatine area ratios (Cho/Cr area) in individuals with blast-related TBI (TBIblast) relative to unexposed controls (non-TBI non-blast). Figure 2B,C show that the blast-induced TBI subgroup exhibits a significantly lower Cho/Cr area ratio compared to the non-blast impact subgroup and non-TBI non-blast controls over the right fronto-parietal white matter and right caudal anterior cingulate, respectively (Table 3).

Superior Frontal and Precentral Cortex: The left superior frontal cortex demonstrated the largest statistical effect (Estimate = −0.0941, SE = 0.0176, t = −5.35, pBonf < 0.0001, Cohen’s d = −0.44, 95% CI [−0.1287, −0.0596]). Corresponding reductions were observed in the right superior frontal cortex (d = −0.34, pBonf = 0.0010), left precentral cortex (d = −0.34, pBonf = 0.0044), and right precentral cortex (d = −0.30, pBonf = 0.0410).

Cerebral White Matter: Bilateral reductions in Cho/Cr area were observed in cerebral white matter for the TBIblast group: Right Cerebral White Matter (Estimate = −0.0895, t = −4.91, pBonf < 0.0001, d = −0.39) and Left Cerebral White Matter (Estimate = −0.0744, t = −4.28, pBonf = 0.0005, d = −0.34).

Parietal and Cingulate Regions: Significant Cho/Cr area decreases in TBIblast vs. controls spanned the right precuneus (d = −0.38, pBonf = 0.0001), left posterior cingulate (d = −0.35, pBonf = 0.0003), right caudal anterior cingulate (d = −0.32, pBonf = 0.0023), and right superior parietal cortex (d = −0.31, pBonf = 0.0043).

Corpus Callosum Metabolite Dynamics

In contrast to the cortical reductions seen in blast TBI, the mid-body (central) corpus callosum (CC) exhibited relative elevations in metabolite ratios among non-blast TBI (TBInonblast) and non-TBI blast (nonTBIblast) groups when compared to non-TBI controls without previous blast exposure:

N-Acetylaspartate Ratios (NAA/Cr): non-TBI with previous blast exposure participants exhibited significant increases in NAA/Cr area (Estimate = 0.6506, t = 3.58, pBonf =0.0097, d = 0.38) and NAA/Cr peak height (Estimate = 0.5303, t = 4.11, pBonf = 0.0012, d = 0.45) than non-TBI controls without previous blast exposure (Figure 2D). Similarly, non-blast TBI displayed an elevated NAA/Cr area ratio (Estimate = 0.2781, t = 3.37, pBonf = 0.0208, d = 0.36) (Figure 2D) and NAA/Cr peak height (Estimate = 0.2175, d = 0.39, pBonf = 0.0087).

Direct Group Comparison: When comparing TBIblast directly against nonTBIblast in the central corpus callosum, TBIblast displayed significantly lower NAA/Cr peak height levels (Estimate = −0.3885, t = −3.24, pBonf =0.0326, d = −0.35).

Direct TBI Modality Contrasts

In direct comparisons between TBI mechanisms (TBIblast vs. TBInonblast), blast-associated TBI showed greater reductions in Cho/Cr area within the right caudal anterior cingulate (Estimate = −0.0597, t = −3.66, pBonf =0.0060, d = −0.30) and right paracentral cortex (Estimate = −0.0443, t = −3.21, pBonf =0.0310, d = −0.26).

Male-Only Sensitivity Analysis: NAA/Cr Findings

Control (blast) vs. Control (non-blast): Remains statistically significant in the male-only cohort for both NAA/Cr area (*p* = 0.0179) and NAA/Cr peak (*p* = 0.0026), demonstrating higher levels in blast-exposed controls.

Preserved Blast-Related Differences: TBI (blast) vs. Control (blast): Remains statistically significant in the male-only cohort for NAA/Cr peak (*p* = 0.0315), showing lower levels in the TBI blast group compared to control blast.

Differences Lost in Male-Only Subset: TBI (non-blast) vs. Control (non-blast): Significant in the primary analysis for both area (*p* = 0.0208) and peak (*p* = 0.0087), but no longer reaches Bonferroni-corrected significance in the male-only sensitivity subset.

Male-Only Sensitivity Analysis across blast TBI subgroups and non-TBI

In the sensitivity analysis restricted to male participants, 12 regional Cho/Cr area differences remained statistically significant after Bonferroni correction. Ten (10) of the 12 regions overlapped with findings from the primary analysis (including right precuneus, bilateral cerebral white matter, left superior frontal cortex, left posterior cingulate, left precuneus, left paracentral/precentral regions, right postcentral cortex, and right posterior cingulate). The right superior parietal and right caudal middle frontal findings from the primary analysis were no longer significant after Bonferroni correction in the male-only analysis. Conversely, bilateral caudal anterior cingulate differences reached Bonferroni-corrected significance in the male-only analysis.

Importantly, all significant group differences in the male-only analysis were in the same direction as the primary findings, with lower Cho/Cr in the mTBI group. These results indicate that the principal pattern of reduced regional Cho/Cr in mTBI was substantially preserved after restricting the analysis to male participants (Tables S2–S6).

Multiple Blast TBI vs. Non-, single Blast Trauma

Stratification of the clinical cohort based on the mechanism of injury unveiled distinct localized metabolic variations, suggesting different biomechanical signatures in repeated blast exposures. Individuals with multiple blast exposures demonstrated a greater reduction in Cho/Cr area ratios than non-TBI at the regions where blast TBI had a reduction in Cho/Cr, i.e., left superior frontal gyrus (Estimate = −0.0791, SE = 0.0166, t = −4.76, pBonf < 0.0001, Cohen’s d = −0.39, 95% CI [−0.1118, −0.0465], right precuneus (Estimate = −0.0841, SE = 0.0180, t = −4.67, pBonf < 0.0001, Cohen’s d = −0.39, 95% CI [−0.1195, −0.0487]), and bilateral fronto-parietal WM (right: Estimate = −0.0840, SE = 0.0172, t = −4.89, pBonf < 0.0001, Cohen’s d = −0.39, 95% CI [−0.1178, −0.0503]; left: Estimate = −0.0769, SE = 0.0163, t = −4.72, Cohen’s d = −0.38, pBonf < 0.0001, 95% CI [−0.1089,−0.0449]). This suggests that the global fronto-parietal reductions in Cho/Cr ratios were found to be predominantly driven by the multiple blast-induced TBI subgroup. In addition, TBI individuals with multiple blast exposures demonstrated significantly lower Cho/Cr values than single-, non-blast TBI at right caudal anterior cingulate (Estimate = −0.0527, SE = 0.0137, t = −3.86, pBonf = 0.0028, Cohen’s d = −0.32, 95% CI [−0.0796, −0.0259], left fronto-parietal WM (Estimate = −0.0383, SE = 0.0108, t = −3.53, pBonf = 0.0099, Cohen’s d = −0.28, 95% CI [−0.0596, −0.0170]), right para-central gyrus (Estimate = −0.0383, SE = 0.0108, t = −3.53, pBonf = 0.0099, Cohen’s d = −0.28, 95% CI [−0.0632, −0.0177]), and right posterior cingulate (Estimate = −0.0387, SE = 0.0115, t = −3.36, pBonf = 0.0180, Cohen’s d = −0.27, 95% CI [−0.0613, −0.0161]) (Table 4).

**Table 3:** MRS Metabolite Differences Across Blast, Non-blast Subgroups and Regions. Post hoc Bonferroni-corrected linear model estimates, standard errors, t-values, and effect sizes (Cohen’s d) of regional brain metabolite changes among blast subgroups within TBI and non-TBI.

Metabolite Measure	Brain Region	Group 1	Group 2 (Control)	Estimate	StdErr	t-Value	Bonferroni p	Cohen’s d	95% CI
Cho/Cr area	Lh superior frontal	TBIblast	nonTBInonblast	−0.0941	0.0176	−5.35	<0.0001	−0.44	[−0.1287, −0.0596]
Cho/Cr area	Rh fronto-parietal white matter	TBIblast	nonTBInonblast	−0.0895	0.0182	−4.91	<0.0001	−0.39	[−0.1253, −0.0537]
Cho/Cr area	Rh precuneus	TBIblast	nonTBInonblast	−0.0900	0.0193	−4.66	0.0001	−0.38	[−0.1279, −0.0521]
Cho/Cr area	Rh postcentral	TBIblast	nonTBInonblast	−0.1033	0.0233	−4.43	0.0003	−0.42	[−0.1491, −0.0575]
Cho/Cr area	Lh posterior cingulate	TBIblast	nonTBInonblast	−0.0815	0.0187	−4.36	0.0003	−0.35	[−0.1182, −0.0448]
Cho/Cr area	Lh fronto-parietal white matter	TBIblast	nonTBInonblast	−0.0744	0.0174	−4.28	0.0005	−0.34	[−0.1085, −0.0403]
Cho/Cr peak height	Midbody corpus callosum	TBInonblast	nonTBInonblast	0.1653	0.0391	4.23	0.0007	0.47	[0.0884, 0.2421]
Cho/Cr area	Rh superior frontal	TBIblast	nonTBInonblast	−0.0741	0.0181	−4.10	0.0010	−0.34	[−0.1096, −0.0386]
NAA/Cr peak height	Midbody corpus callosum	nonTBIblast	nonTBInonblast	0.5303	0.1289	4.11	0.0012	0.45	[0.2767, 0.7839]
Cho/Cr area	Lh precuneus	TBIblast	nonTBInonblast	−0.0786	0.0199	−3.96	0.0019	−0.32	[−0.1176, −0.0396]
Cho/Cr area	Lh paracentral	TBIblast	nonTBInonblast	−0.0707	0.0180	−3.93	0.0020	−0.32	[−0.1061, −0.0354]
Cho/Cr area	Rh caudal anterior cingulate	TBIblast	nonTBInonblast	−0.0865	0.0222	−3.90	0.0023	−0.32	[−0.1301, −0.0430]
Cho/Cr area	Rh posterior cingulate	TBIblast	nonTBInonblast	−0.0724	0.0189	−3.83	0.0031	−0.31	[−0.1096, −0.0353]
Cho/Cr area	Rh superior parietal	TBIblast	nonTBInonblast	−0.0793	0.0212	−3.75	0.0043	−0.31	[−0.1209, −0.0377]
Cho/Cr area	Lh precentral	TBIblast	nonTBInonblast	−0.0732	0.0195	−3.74	0.0044	−0.34	[−0.1116, −0.0348]
Cho/Cr area	Rh caudal anterior cingulate	TBIblast	TBInonblast	−0.0597	0.0163	−3.66	0.0060	−0.30	[−0.0917, −0.0277]
NAA/Cr peak height	Midbody corpus callosum	TBInonblast	nonTBInonblast	0.2175	0.0602	3.61	0.0087	0.39	[0.0991, 0.3360]
NAA/Cr area	Midbody corpus callosum	nonTBIblast	nonTBInonblast	0.6506	0.1817	3.58	0.0097	0.38	[0.2933, 1.0080]
Cho/Cr area	Rh paracentral	TBIblast	nonTBInonblast	−0.0651	0.0185	−3.52	0.0104	−0.28	[−0.1015, −0.0287]
Cho/Cr area	Rh postcentral	TBInonblast	nonTBInonblast	−0.0927	0.0267	−3.47	0.0127	−0.33	[−0.1453, −0.0402]
NAA/Cr area	Midbody corpus callosum	TBInonblast	nonTBInonblast	0.2781	0.0825	3.37	0.0208	0.36	[0.1159, 0.4403]
Cho/Cr area	Rh postcentral	nonTBIblast	nonTBInonblast	−0.1827	0.0563	−3.24	0.0280	−0.31	[−0.2934, −0.0720]
Cho/Cr area	Rh caudal middle frontal	TBIblast	nonTBInonblast	−0.0715	0.0222	−3.22	0.0297	−0.28	[−0.1151, −0.0279]
Cho/Cr area	Rh paracentral	TBIblast	TBInonblast	−0.0443	0.0138	−3.21	0.0310	−0.26	[−0.0714, −0.0172]
NAA/Cr peak height	Midbody corpus callosum	TBIblast	nonTBIblast	−0.3885	0.1198	−3.24	0.0326	−0.35	[−0.6242, −0.1528]
Cho/Cr peak height	Midbody corpus callosum	nonTBIblast	nonTBInonblast	0.2603	0.0809	3.22	0.0341	0.36	[0.1012, 0.4194]
Cho/Cr peak height	Lh superior frontal	TBIblast	nonTBInonblast	−0.0495	0.0156	−3.17	0.0379	−0.26	[−0.0802, −0.0189]
Cho/Cr area	Lh caudal anterior cingulate	TBIblast	nonTBInonblast	−0.0693	0.0221	−3.13	0.0399	−0.25	[−0.1128, −0.0259]
Cho/Cr area	Rh precentral	TBIblast	nonTBInonblast	−0.0647	0.0207	−3.13	0.0410	−0.30	[−0.1053, −0.0241]

**Table 4:** MRS Metabolite Differences Across Multiple Blast, Single/Non-blast Subgroups and Regions. Post hoc Bonferroni-corrected linear model estimates, standard errors, t-values, and effect sizes (Cohen’s d) of regional brain metabolite changes among multiple blast exposure, single/non-blast subgroups within TBI versus non-TBI.

Metabolite Measure	Brain Region	Group 1	Group 2 (Control)	Estimate	StdErr	t-Value	Bonferroni *p*	Cohen’s d	95% CI
Cho/Cr area	Rh fronto-parietal white matter	MultipleBlasts	nonTBI	−0.0840	0.0172	−4.89	<0.0001	−0.39	[−0.1178, −0.0503]
Cho/Cr area	Lh superior frontal	MultipleBlasts	nonTBI	−0.0791	0.0166	−4.76	<0.0001	−0.39	[−0.1118, −0.0465]
Cho/Cr area	Lh fronto-parietal white matter	MultipleBlasts	nonTBI	−0.0769	0.0163	−4.72	<0.0001	−0.38	[−0.1089, −0.0449]
Cho/Cr area	Rh precuneus	MultipleBlasts	nonTBI	−0.0841	0.0180	−4.67	<0.0001	−0.39	[−0.1195, −0.0487]
Cho/Cr area	Lh posterior cingulate	MultipleBlasts	nonTBI	−0.0795	0.0174	−4.56	0.00013	−0.37	[−0.1137, −0.0453]
Cho/Cr area	Lh paracentral	MultipleBlasts	nonTBI	−0.0684	0.0168	−4.07	0.00116	−0.33	[−0.1015, −0.0354]
Cho/Cr area	Lh precuneus	MultipleBlasts	nonTBI	−0.0746	0.0186	−4.02	0.00147	−0.33	[−0.1111, −0.0381]
Cho/Cr area	Lh precentral	MultipleBlasts	nonTBI	−0.0717	0.0182	−3.94	0.00204	−0.36	[−0.1074, −0.0360]
Cho/Cr area	Rh posterior cingulate	MultipleBlasts	nonTBI	−0.0688	0.0177	−3.89	0.00246	−0.32	[−0.1035, −0.0340]
Cho/Cr area	Rh caudal anterior cingulate	MultipleBlasts	NonMultipleBlast	−0.0527	0.0137	−3.86	0.00281	−0.32	[−0.0796, −0.0259]
Cho/Cr area	Rh caudal anterior cingulate	MultipleBlasts	nonTBI	−0.0780	0.0210	−3.71	0.00496	−0.30	[−0.1193, −0.0367]
Cho/Cr area	Rh paracentral	MultipleBlasts	nonTBI	−0.0630	0.0174	−3.62	0.00705	−0.29	[−0.0971, −0.0288]
Cho/Cr area	Lh fronto-parietal matter	MultipleBlasts	NonMultipleBlast	−0.0383	0.0108	−3.53	0.00986	−0.28	[−0.0596, −0.0170]
Cho/Cr area	Rh superior parietal	MultipleBlasts	nonTBI	−0.0697	0.0198	−3.52	0.01011	−0.29	[−0.1086, −0.0309]
Cho/Cr area	Rh superior frontal	MultipleBlasts	nonTBI	−0.0600	0.0171	−3.52	0.01029	−0.29	[−0.0935, −0.0265]
Cho/Cr area	Rh postcentral	MultipleBlasts	nonTBI	−0.0770	0.0220	−3.50	0.01119	−0.33	[−0.1203, −0.0338]
Cho/Cr area	Rh paracentral	MultipleBlasts	NonMultipleBlast	−0.0404	0.0116	−3.49	0.01152	−0.28	[−0.0632, −0.0177]
Cho/Cr area	Rh posterior cingulate	MultipleBlasts	NonMultipleBlast	−0.0387	0.0115	−3.36	0.01800	−0.27	[−0.0613, −0.0161]
Cho/Cr area	Rh caudal middle frontal	MultipleBlasts	nonTBI	−0.0690	0.0211	−3.27	0.02520	−0.29	[−0.1105, −0.0276]
Cho/Cr area	Rh precuneus	NonMultipleBlast	nonTBI	−0.0630	0.0193	−3.26	0.02602	−0.27	[−0.1009, −0.0250]
Cho/Cr area	Lh caudal anterior cingulate	MultipleBlasts	nonTBI	−0.0673	0.0208	−3.24	0.02807	−0.27	[−0.1081, −0.0265]
Cho/Cr area	Lh caudal middle frontal	MultipleBlasts	nonTBI	−0.0566	0.0178	−3.17	0.03505	−0.27	[−0.0916, −0.0216]
Cho/Cr area	Lh posterior cingulate	MultipleBlasts	NonMultipleBlast	−0.0349	0.0114	−3.07	0.04997	−0.25	[−0.0572, −0.0125]

Male-Only Sensitivity analysis: Fifteen (15) regions were significant in both analyses, 2 were significant only in the primary analysis, and 1 was significant only in the male-only analysis. All summarized significant effects were in the direction of a lower Cho/Cr area in Multiple-Blast TBI relative to non-TBI controls (Table S7).

Associations between brain metabolites and behavioral measures

To determine the functional relevance of these metabolic patterns, Pearson partial correlation analyses were performed controlling for age and gender. Chronicity of the injury (measured as days elapsed since index trauma) demonstrated no significant correlations with any regional metabolic ratios (*p* > 0.05), indicating that these metabolic alterations remain relatively stable throughout the chronic phase.

**Figure 3:**
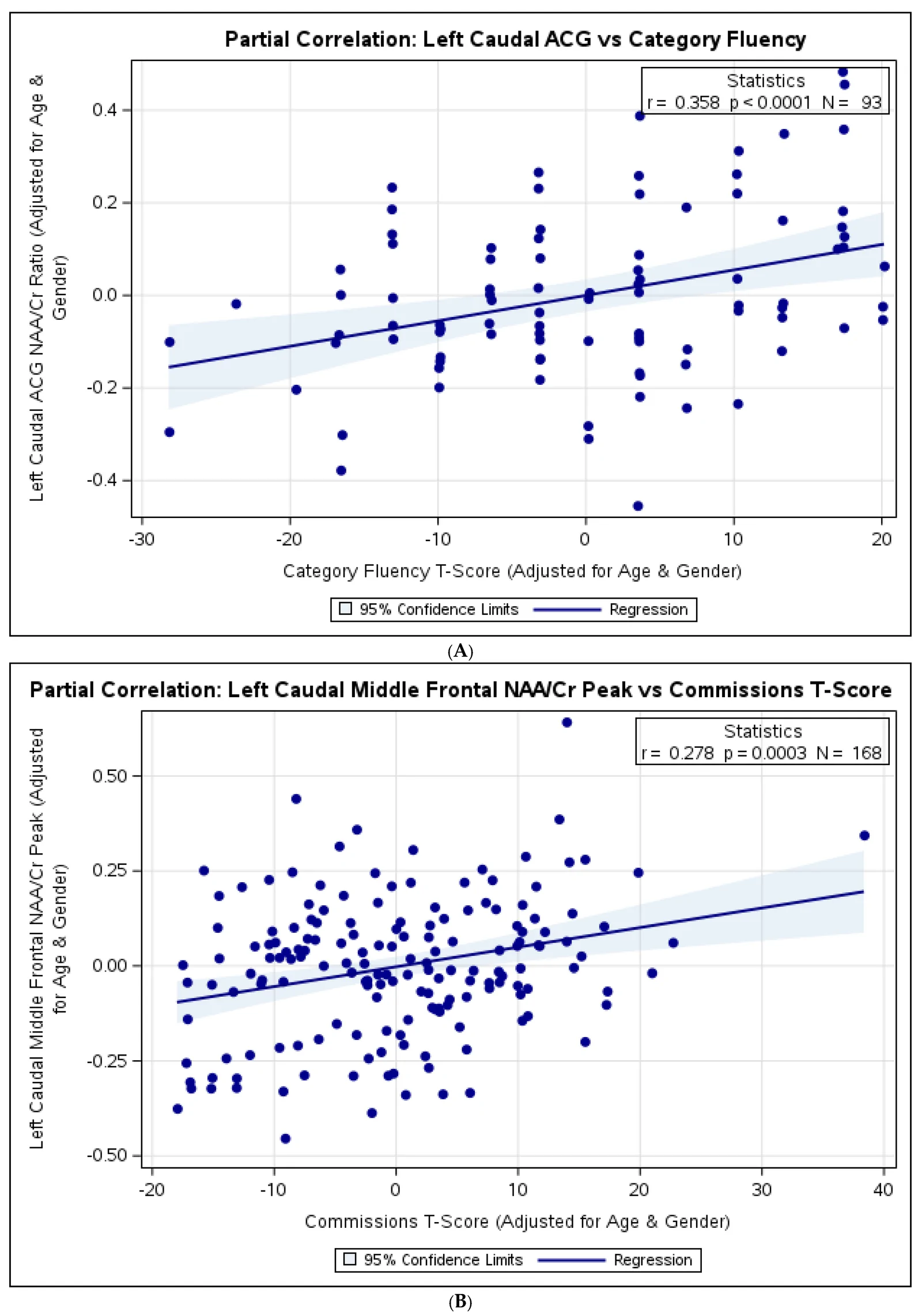

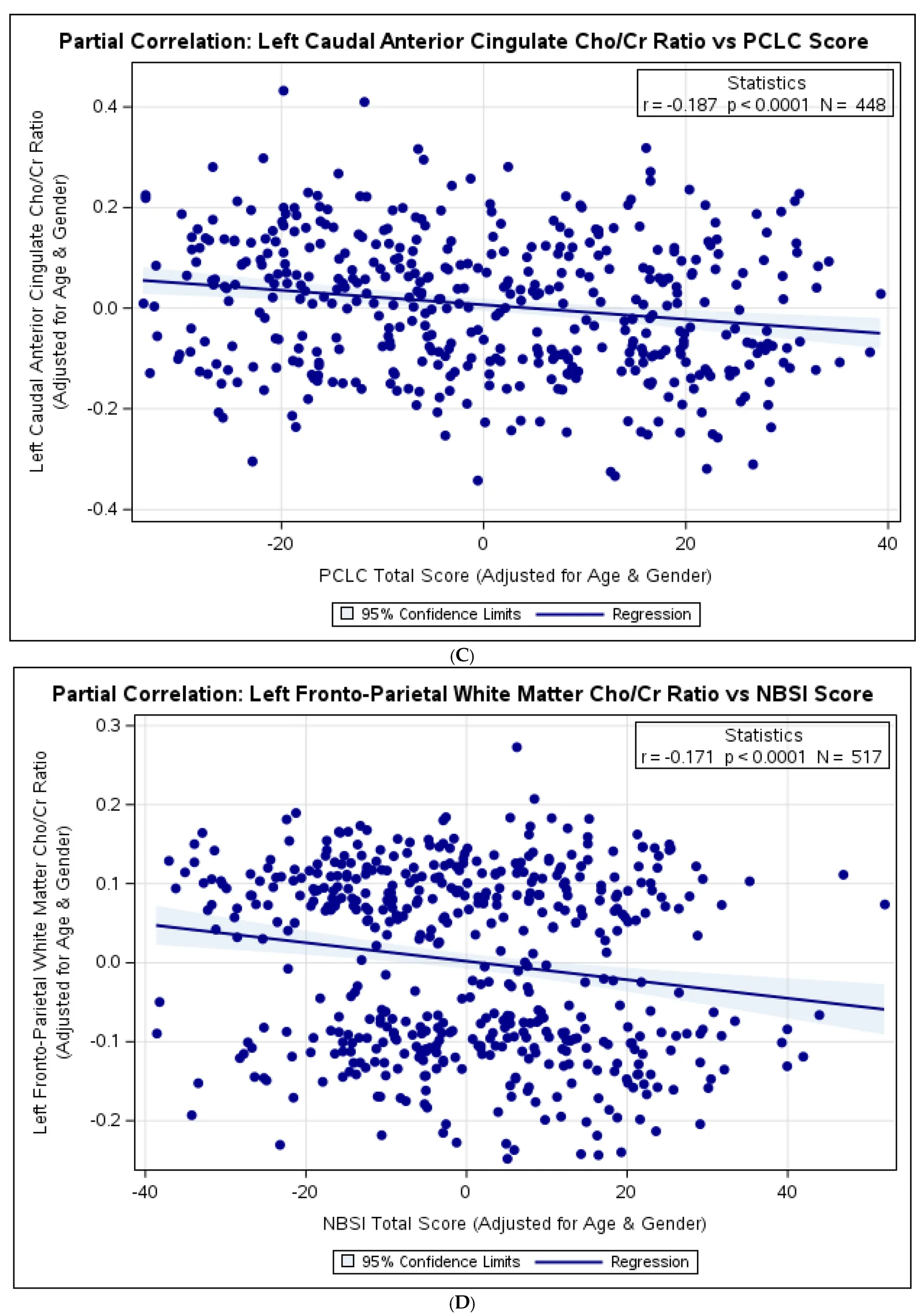
**Partial Correlation Scatterplots Mapping Fronto-parietal Metabolite Integrity to Neurocognitive Efficiency and Post-Concussive Symptoms (total NeuroBehavioral Symptom Inventory score).** Adjusted partial correlation scatterplots (controlling for age and gender as fixed covariates) illustrating the translational clinical relevance of localized metabolic ratios. (**A**) Mean NAA/Cr area over the left caudal anterior cingulate (ACG) correlates positively with verbal fluency performance (Category Fluency t-score) (r = 0.358, *p* < 0.0001). (**B**) Response inhibition accuracy (Commissions t-score) positively correlated with NAA/Cr peak height in the left caudal middle frontal cortex (r = 0.278, *p* = 0.0003). (**C**) Lower Cho/Cr was significantly associated with higher PCL-C total scores in several regions in the left caudal anterior cingulate gyrus (r = −0.187, *p* < 0.0001). (**D**) Cho/Cr in the left fronto-parietal white matter was inversely associated with neurobehavioral symptom severity, assessed by the Neurobehavioral Symptom Inventory (NSI) (r = −0.171, *p* < 0.0001).

Significant associations were identified between regional brain metabolite measures and post-traumatic stress symptoms, neurobehavioral symptoms, and cognitive performance in participants with military-related TBI (Table 5). Specifically, associations involving N-acetylaspartate-to-creatine (NAA/Cr) measures showed a different pattern and were primarily related to cognitive performance. NAA/Cr area in the left caudal anterior cingulate gyrus was positively associated with Category Fluency T-score (estimate = 0.00550, SE = 0.00152, t = 3.61, *p* < 0.0001, 95% CI [0.00247, 0.00852]) (Figure 3A). In addition, NAA/Cr peak height in the left caudal middle frontal gyrus was positively associated with performance on the Commissions T-score measure (estimate = 0.00516, SE = 0.00138, t = 3.74, *p* = 0.0003, 95% CI [0.00243, 0.00789]) (Figure 3B). A comparable positive association was observed for NAA/Cr peak height in the left caudal anterior cingulate gyrus and Category Fluency T-score (estimate = 0.00416, SE = 0.00118, t = 3.54, *p* = 0.00065, 95% CI [0.00182, 0.00649]).

The most consistent pattern involved the choline-to-creatine (Cho/Cr) ratio, which was inversely associated with post-traumatic stress symptom severity as measured by the Posttraumatic Stress Checklist–Civilian Version (PCL-C) total score. Lower Cho/Cr was significantly associated with higher PCL-C total scores in several regions. The strongest association was observed in the left caudal anterior cingulate gyrus (estimate = −0.00144, SE = 0.00036, t = −4.02, *p* < 0.0001, 95% CI [−0.00215, −0.00074]) (Figure 3C). Similar inverse associations were observed in the right cerebral white matter (estimate = −0.00124, SE = 0.00031, t = −4.01, *p* < 0.0001, 95% CI [−0.00185, −0.00063]) and left cerebral white matter (estimate = −0.00115, SE = 0.00030, t = −3.87, *p* = 0.00012, 95% CI [−0.00174, −0.00057]). An inverse association was also observed in the left posterior cingulate gyrus (estimate = −0.00115, SE = 0.00031, t = −3.76, *p* = 0.00019, 95% CI [−0.00175, −0.00055]). The association between Cho/Cr and PCL-C scores extended to bilateral precuneus regions. Specifically, Cho/Cr was inversely associated with PCL-C total score in the left precuneus (estimate = −0.00120, SE = 0.00033, t = −3.62, *p* = 0.00033, 95% CI [−0.00186, −0.00055]) and right precuneus (estimate = −0.00116, SE = 0.00032, t = −3.60, *p* = 0.00035, 95% CI [−0.00179, −0.00053]). A significant inverse association was also observed between Cho/Cr and PCL-C score in the right posterior cingulate gyrus (estimate = −0.00109, SE = 0.00031, t = −3.47, *p* = 0.00056, 95% CI [−0.00170, −0.00047]). These findings indicate a spatially distributed relationship between lower Cho/Cr measures and greater post-traumatic stress symptom burden involving anterior and posterior cingulate, white matter, and precuneus regions.

Cho/Cr was additionally associated with neurobehavioral symptom severity as assessed by the Neurobehavioral Symptom Inventory (NSI). Significant inverse associations were identified in the left cerebral white matter (estimate = −0.00117, SE = 0.00030, t = −3.92, *p* < 0.0001, 95% CI [−0.00176, −0.00058]) (Figure 3D) and right cerebral white matter (estimate = −0.00117, SE = 0.00031, t = −3.73, *p* = 0.00022, 95% CI [−0.00178, −0.00055]). A similar association was observed in the right precuneus (estimate = −0.00109, SE = 0.00032, t = −3.35, *p* = 0.00086, 95% CI [−0.00172, −0.00045]).

## 4. Discussion

The principal findings reveal: (1) extensive, pervasive reductions in the Cho/Cr area across several supracallosal fronto-parietal regions in the TBI cohort; (2) blast-associated TBI causes widespread reductions in cortical and subcortical Cho/Cr area; and (3) significant, regional Cho/Cr and NAA/Cr directly track clinical neurobehavioral symptom severity and executive functioning performance. Crucially, the absence of a correlation between these metabolic metrics and injury chronicity suggests that these neurochemical alterations reflect stable, persistent microstructural adjustments or permanent cellular damage rather than transient post-concussive disruptions [23].

Pathophysiological Implications of Regional Metabolic Alterations

The direction of the Cho/Cr finding is not inconsistent with the heterogeneous TBI MRS literature. Choline has been associated with membrane phospholipid metabolism and turnover [21], but its response varies across injury severity, region, timing, and methodology. In the acute to subacute phases of TBI, the literature frequently documents elevations in Cho, reflecting accelerated membrane breakdown and cellular shear-induced tearing. Prior work has reported both increased [15,24] and decreased Cho/Cr [19] after TBI, including decreases during the late-subacute phase in some regions [19]. The present cross-sectional findings therefore add another regional pattern to the literature rather than establishing a uniform biochemical response.

NAA/Cr showed a different pattern from Cho/Cr in the exploratory clinical analyses. NAA is frequently interpreted as a marker related to neuronal metabolism [25,26], but its signal reflects more than a single cellular compartment and should not be equated with irreversible axonal loss.

Corpus Callosum Compensation vs. Vulnerability

The central corpus callosum demonstrated dynamic metabolic shifts. Elevated NAA/Cr and Cho/Cr ratios in non-blast TBI (d = 0.39 to 0.47) and non-TBI blast exposures (d = 0.38 to 0.45) indicate secondary compensatory metabolic mechanisms or localized axonal repair processes. However, when blast exposure coincides with mechanical TBI (TBIblast), this callosal compensation is blunted, as demonstrated by the relative decrease in callosal NAA/Cr peak height compared to blast exposure alone (d = −0.35).

Sex Imbalance and Sensitivity Analysis

The male-only sensitivity analysis provides useful context for the substantial sex imbalance in the cohorts. Most primary regional Cho/Cr findings remained significant and in the same direction after restricting the analysis to males, although some regions changed significance and additional caudal anterior cingulate findings emerged. This supports the robustness of the broad group pattern to one aspect of the cohort imbalance, but it does not eliminate other potential sources of confounding. Nevertheless, the small number of female participants in the mTBI group limits the ability to assess sex-specific effects and underscores the need for adequately powered studies examining sex as a potential modifier of regional metabolic abnormalities after TBI.

Mechanistic Signatures: Blast vs. Non-Blast Trauma

A central focus of military clinical neuroscience is determining whether blast-induced neurotrauma represents a separate pathophysiological entity from impact-driven injuries [27]. Our subgroup analyses reveal a compelling neurochemical divergence that supports distinct mechanical signatures. First, the widespread reductions in Cho/Cr across the left frontal white matter were predominantly driven by the blast-induced TBI cohort, suggesting that blast waves induce a more generalized, pervasive form of microstructural white matter strain than localized blunt impacts.

The six non-TBI controls with remote operational blast exposure means that a nominal non-TBI control group is not completely homogeneous with respect to blast history. Findings involving these participants may reflect exposure history, other between-group differences, or sampling variability.

Repeated blast exposure and Cho/Cr

The association between Cho/Cr and symptom measures may also be relevant to the heterogeneous injury histories commonly observed in military populations. In the present cohort, individuals with multiple blast exposures demonstrated a greater reduction in Cho/Cr area ratios than individuals with non-blast or single-blast TBI. This pattern suggests a possible exposure-related gradient in regional membrane-associated metabolic measures. To our knowledge, the existing human MRS literature does not yet provide sufficient evidence to establish a reproducible dose–response relationship between the number of blast exposures and Cho/Cr. Accordingly, the present finding should be considered an observation requiring replication rather than as confirmation of an established MRS dose–response relationship. Nevertheless, the possibility that repeated blast exposure is associated with greater regional metabolic alteration is biologically plausible and warrants investigation in larger military cohorts with detailed exposure histories.

Implications of Choline Reductions in Blast TBI

The concentration of peak effect sizes in the superior frontal (d = −0.44) and postcentral cortices (d = −0.42) aligns with biomechanical models showing that primary blast overpressure waves concentrate shear stress across superficial frontal and parietal cortical boundaries. While elevated Cho/Cr is classically linked to active neuroinflammation and reactive gliosis due to increased glial membrane synthesis, the widespread reduction in Cho/Cr observed here likely reflects impaired cell membrane turnover, reduced glial cell density, or chronic neurovascular structural degradation secondary to blast exposure. Interestingly, our concurrent diffusion MRI findings, employing neurite orientation dispersion and density imaging and fixel-based analysis, demonstrate persistent neuroinflammation and reactive gliosis [28]. MRS reveals a reduction in Cho/Cr ratios across several cortical and white matter regions. This apparent divergence underscores the distinct biological processes captured by these modalities. The low Cho/Cr reflects suppressed membrane remodeling or altered intracellular choline storage rather than a complete absence of glial cells. Military injuries can involve blast, impact, falls, and other mechanisms simultaneously, so these contrasts cannot be interpreted as direct measures of biomechanical strain or as evidence that blast produces a unique metabolic phenotype. The multiple-blast subgroup showed lower Cho/Cr than controls across several regions, with the most significant findings retained in a male-only sensitivity analysis.

Metabolic Correlates of Neurocognitive and Psychological Dysfunction

The present findings demonstrate significant relationships between regional brain metabolite measures and behavioral outcomes in individuals with military-related TBI. Two principal patterns emerged. First, higher NAA/Cr was associated with better performance on selected measures of executive and language-related cognitive functioning. Second, lower Cho/Cr was associated with greater post-traumatic stress symptom severity across multiple cortical and white matter regions.

The exploratory clinical associations provide a second line of evidence linking regional metabolite measures with symptom and cognitive variation. Higher selected NAA/Cr measures were associated with better performance on Category Fluency and Commissions measures, whereas lower Cho/Cr was associated with greater PCL-C and NSI symptom burden. These associations are compatible with prior reports that MRS measures may vary with clinical and cognitive outcomes after TBI [15,19,29]. The involvement of the caudal middle frontal and anterior cingulate regions is also compatible with the established involvement of frontal and anterior cingulate systems in executive control, cognitive control, response monitoring, attention, and higher-order cognitive functioning [30].

The distribution of PCL-C associations across cingulate, precuneus, and white-matter regions is anatomically interesting, but it should not be interpreted as evidence of altered functional connectivity or a specific default-mode-network mechanism. Multimodal studies combining MRSI with structural, diffusion, and functional imaging could test whether regional metabolic variation covaries with independently measured network or microstructural abnormalities.

Furthermore, the correlations do not establish causality, and the biological meaning of ratio measures remains dependent on both numerator and denominator. The relationships may also be influenced by injury characteristics, premorbid factors, medication exposure, comorbid symptoms, and other clinical variables.

Limitations and Future Directions

While this study boasts an exceptionally large military sample size, several limitations must be acknowledged. First, the cross-sectional design prevents the direct tracking of individual longitudinal metabolic trajectories. Longitudinal follow-up studies are required to determine whether these chronic fronto-parietal metabolic alterations represent stabilized scarring, or if they continuously evolve over years. Second, the demographic composition of our sample was overwhelmingly male (>95%), reflecting deployment statistics of the era, which limits the direct generalizability of these findings to female service members or civilian TBI populations. Finally, while multi-voxel 2D ^1^H-MRSI provides excellent coverage across a single supracallosal plane, it leaves out deeper subcortical structures and brainstem nuclei that are also highly susceptible to diffuse axonal injury; future studies should employ whole-brain 3D MRSI techniques to capture a global neurochemical map.

Although the MRSI data underwent standardized reconstruction and processing, standardized retrospective linewidth/FWHM and motion-exclusion metrics were not available, nor was formal voxel-wise tissue-fraction correction performed. Regional measures were derived after anatomical registration and regional averaging. In addition, multiple metabolite measures, regions, peak-height/area metrics, and clinical outcomes were examined; clinical correlations are therefore treated as exploratory rather than validated clinical biomarkers.

Finally, the study was not designed to measure pressure waves, shear strain, cavitation, or other biomechanical quantities. Blast exposure may occur together with impact and other forms of trauma, and the clinical classification of mechanism is based on exposure history rather than a direct physical measurement. Accordingly, the observed regional differences can identify statistical contrasts between exposure-defined groups but cannot establish a unique biochemical signature of blast or a dose–response relation with the number of exposures.

## 5. Conclusions

In this military TBI cohort, ^1^H-MRSI demonstrated regional differences in NAA/Cr and Cho/Cr measures, with lower Cho/Cr observed across multiple supracallosal fronto-parietal regions. The broad pattern was substantially preserved in a male-only sensitivity analysis. Blast- and multiple-blast subgroup analyses identified additional regional differences, but these findings remain hypothesis-generating because injury mechanisms may overlap. Exploratory associations linked selected NAA/Cr measures with cognitive performance and lower Cho/Cr with greater PCL-C and NSI symptom burden. Overall, the data support the presence of regional metabolite-ratio differences after military-related TBI, particularly lower Cho/Cr in several fronto-parietal regions, together with exploratory associations between metabolite ratios and clinical measures. These cross-sectional findings describe regional metabolic correlates; they do not establish diagnostic sensitivity, cellular mechanisms, biomechanical strain patterns, causality, or longitudinal recovery. The results provide a basis for future studies that combine MRSI with structural and diffusion imaging, functional measures, and detailed longitudinal exposure histories.

## Figures and Tables

**Table 1:** Participant Demographics and Clinical Characteristics.

Demographic/Clinical Metric	Non-TBI Controls (n = 75)	Blast Non-TBI Subgroup (n = 6)	Non-Blast Non-TBI Subgroup (n = 69)	Total TBI Cohort (n = 737) Severe (n = 11) Moderate (n = 48) Mild (n = 678	Blast-Induced TBI Subgroup (n = 535)	Blunt/Non-Blast TBI Subgroup (n = 206)
Age (years, Mean ± SD)	33.1 ± 10.0	25.3 ± 6.6	33.8 ± 10.0	34.3 ± 8.1	33.8 ± 7.7	35.7 ± 8.9
Gender (Male/Female)	53/22	6/0	47/22	711/30	523/12	188/18
Days Since Injury (Mean ± SD) Range	N/A	N/A	N/A	327.4 ± 226.8 [29, 996]	338.1 ± 228.3 [29, 996]	303.4 ± 223.7 [34, 933]
NSI Total Score (Mean ± SD)	6.2 ± 9.3	4.6 ± 5.9	6.4 ± 9.7	29.5 ± 41.2 *	30.3 ± 14.3 *	27.2 ± 74.8 *
PCL-C Total Score (Mean ± SD)	22.5± 9.4	24.0 ± 4.5	22.3± 9.8	48.7 ± 19.1 *	52.0 ± 18.5 *	38.4 ± 17.3 *

Note: NSI = Neurobehavioral Symptom Inventory; PCL-C = Post-Traumatic Stress Disorder Checklist–Civilian version. * indicates significantly different from Non-TBI controls (*p* < 0.05, Bonferroni corrected).

**Table 2:** Summary of Regional Group Differences in Fronto-parietal Metabolites: TBI vs. non-TBI Group Differences.

Region	Estimate	StdErr	t-Value	*p*-Value Bonferroni	Cohen’s d	95% CI
Rh precuneus	−0.0781	0.0176	−4.43	2.48 × 10^−4^	−0.364	[−0.1127, −0.0435]
Rh fronto-parietal white matter	−0.0741	0.0169	−4.39	2.88 × 10^−4^	−0.350	[−0.1072, −0.0410]
Lh superior frontal	−0.0715	0.0163	−4.38	3.09 × 10^−4^	−0.355	[−0.1035, −0.0394]
Lh fronto-parietal white matter	−0.0656	0.0161	−4.08	0.0011	−0.325	[−0.0971, −0.0340]
Lh posterior cingulate	−0.0690	0.0171	−4.03	0.0014	−0.325	[−0.1027, −0.0354]
Lh precuneus	−0.0677	0.0182	−3.72	0.0048	−0.304	[−0.1034, −0.0319]
Lh paracentral	−0.0604	0.0165	−3.65	0.0062	−0.295	[−0.0929, −0.0280]
Lh precentral	−0.0632	0.0178	−3.56	0.0090	−0.322	[−0.0982, −0.0283]
Rh postcentral	−0.0727	0.0215	−3.38	0.0174	−0.318	[−0.1150, −0.0304]
Rh superior parietal	−0.0639	0.0193	−3.31	0.0221	−0.273	[−0.1018, −0.0259]
Rh caudal middle frontal	−0.0671	0.0205	−3.27	0.0255	−0.287	[−0.1074, −0.0267]
Rh posterior cingulate	−0.0565	0.0174	−3.25	0.0268	−0.262	[−0.0907, −0.0224]

**Table 5:** Associations Between Regional Brain Metabolites and Neurocognitive/Neurobehavioral Symptoms. Linear model estimates regressing out age and gender effects (Metabolite ~ Symptom/Cognitive Score + Age + Gender).

Metabolite Measure	Brain Region	Variable/Scale	Estimate	StdErr	t-Value	*p*-Value	95% Confidence Interval
Cho/Cr area	Lh caudal anterior cingulate	PCLC total	−0.00144	0.00036	−4.02	<0.0001	[−0.00215, −0.00074]
Cho/Cr area	Rh fronto-parietal white matter	PCLC total	−0.00124	0.00031	−4.01	<0.0001	[−0.00185, −0.00063]
Cho/Cr area	Lh fronto-parietal white matter	NSI total	−0.00117	0.00030	−3.92	<0.0001	[−0.00176, −0.00058]
Cho/Cr area	Lh fronto-parietal white matter	PCLC total	−0.00115	0.00030	−3.87	0.00012	[−0.00174, −0.00057]
Cho/Cr area	Lh posterior cingulate	PCLC total	−0.00115	0.00031	−3.76	0.00019	[−0.00175, −0.00055]
Cho/Cr area	Rh fronto-parietal white matter	NSI total	−0.00117	0.00031	−3.73	0.00022	[−0.00178, −0.00055]
NAA/Cr peak height	Lh caudal middle frontal	Commissions t-score	0.00516	0.00138	3.74	0.00026	[0.00243, 0.00789]
Cho/Cr area	Lh precuneus	PCLC total	−0.00120	0.00033	−3.62	0.00033	[−0.00186, −0.00055]
Cho/Cr area	Rh precuneus	PCLC total	−0.00116	0.00032	−3.60	0.00035	[−0.00179, −0.00053]
NAA/Cr area	Lh caudal anterior cingulate	Category Fluency t-score	0.00550	0.00152	3.61	0.00050	[0.00247, 0.00852]
	Rh posterior cingulate	PCLC total	−0.00109	0.00031	−3.47	0.00056	[−0.00170, −0.00047]
NAA/Cr peak height	Lh caudal anterior cingulate	Category Fluency t-score	0.00416	0.00118	3.54	0.00065	[0.00182, 0.00649]
Cho/Cr area	Rh precuneus	NSI total	−0.00109	0.00032	−3.35	0.00086	[−0.00172, −0.00045]

## Data Availability

Summary/aggregate data and additional information on the methods and statistical analyses will be provided on request. However, individual data elements, including individual imaging data, are not available due to DoD legal requirements and current IRB-approved language in the subject consent forms.

## Supplementary Materials

The following supporting information can be downloaded at: https://www.mdpi.com/article/10.3390/brainsci16101088/s1, Table S1: Sensitivity-analysis summary of TBI vs non-TBI control; Table S2. Sensitivity-analysis summary of TBIblast vs TBInonblast; Table S3. Sensitivity-analysis summary of TBIblast vs nonTBInonblast; Table S4. Sensitivity-analysis summary of nonTBIblast vs nonTBInonblast; Table S5. Sensitivity-analysis summary of nonTBIblast vs nonTBInonblast; Table S6. Sensitivity-analysis summary of TBIblast vs nonTBIblast; Table S7. Sensitivity analysis summary of Multiple-blast TBI vs non-TBI control.
