# Supplementary material for: Fronto-Parietal Brain Metabolites Following Traumatic Brain Injury—A Proton MR Spectroscopy Study
Source: Brain Sci. 2026 Oct 9;16(10):1088. doi: 10.3390/brainsci16101088 (PMC13702281; doi:10.3390/brainsci16101088)
Supplement: Supplementary file 1 [file brainsci-16-01088-s001.zip › brainsci-4440945-supplementary.pdf]

## Supplementary Materials

### Sensitivity Analysis of MRSI Findings by Sex

Primary analysis: analysis including both male and female participants. Male-only sensitivity analysis: analysis restricted to male participants to assess whether findings were robust to the sex imbalance between the Multiple-Blast TBI and non-TBI control groups. Overlapping: region significant in both the primary and male-only analyses. Primary analysis only: region significant in the primary analysis but not in the male-only sensitivity analysis. Male-only specific: region significant only in the male-only sensitivity analysis. Direction indicates the direction of the TBI-versus-control difference. Direction refers to the direction of the regional metabolite difference for Multiple-Blast TBI relative to non-TBI controls.

**Statistical criterion.** Rows are included when Bonferroni-adjusted $p < 0.05$ in either the full-cohort or male-only analysis. The corresponding p-value from the other analysis is retained as “Not significant” when it does not meet this threshold.

### TBI vs non-TBI controls

| Region | Primary analysis Bonferroni p | Male-only Bonferroni p | Direction |
|---|---|---|---|
| Right precuneus | 0.000248 | 0.000238 | TBI < control |
| Left superior frontal | 0.000309 | 0.000249 | TBI < control |
| Left cerebral WM | 0.001110 | 0.000487 | TBI < control |
| Right cerebral WM | 0.000288 | 0.000538 | TBI < control |
| Left posterior cingulate | 0.001392 | 0.002603 | TBI < control |
| Right postcentral | 0.017356 | 0.009789 | TBI < control |
| Left precuneus | 0.004848 | 0.012106 | TBI < control |
| Left precentral | 0.009042 | 0.012121 | TBI < control |
| Left paracentral | 0.006168 | 0.030464 | TBI < control |
| Right posterior cingulate | 0.026806 | 0.029871 | TBI < control |
| Right superior parietal | 0.022126 | 0.120560 | TBI < control |
| Right caudal middle frontal | 0.025498 | 0.167314 | TBI < control |
| Left caudal anterior cingulate | 0.072466 | 0.029783 | TBI < control |
| Right caudal anterior cingulate | 0.072466 | 0.043604 | TBI < control |

**Table S1. Sensitivity-analysis summary of TBI vs non-TBI control.** Fifteen (15) regions were significant in both analyses, 2 were significant only in the primary analysis, and 1 was significant only in the male-only analysis. All summarized significant effects were in the direction of lower Cho/Cr area in mTBI relative to non-TBI controls.

## TBIblast vs TBInonblast

| Brain region | MRSI measure | Group contrast | Primary Bonferroni p | Male-only Bonferroni p | Finding | Direction |
|---|---|---|---|---|---|---|
| Left cerebral white matter | Cho/Cr area | TBIblast vs TBInonblast | 0.0614 | 4.18e-06 | Male-only specific | TBIblast < TBInonblast |
| Right cerebral white matter | Cho/Cr area | TBIblast vs TBInonblast | 0.0686 | 1.17e-06 | Male-only specific | TBIblast < TBInonblast |
| Left caudal anterior cingulate | Cho/Cr area | TBIblast vs TBInonblast | 0.0913 | 7.49e-07 | Male-only specific | TBIblast < TBInonblast |
| Left caudal middle frontal | Cho/Cr area | TBIblast vs TBInonblast | 0.1296 | 1.06e-04 | Male-only specific | TBIblast < TBInonblast |
| Left isthmus cingulate | Cho/Cr area | TBIblast vs TBInonblast | Not significant | 0.0446 | Male-only specific | TBIblast < TBInonblast |
| Left paracentral region | Cho/Cr area | TBIblast vs TBInonblast | Not significant | 0.0022 | Male-only specific | TBIblast < TBInonblast |
| Left posterior cingulate | Cho/Cr area | TBIblast vs TBInonblast | Not significant | 3.21e-04 | Male-only specific | TBIblast < TBInonblast |
| Left precuneus | Cho/Cr area | TBIblast vs TBInonblast | Not significant | 5.40e-04 | Male-only specific | TBIblast < TBInonblast |
| Left superior frontal cortex | Cho/Cr area | TBIblast vs TBInonblast | 0.0596 | 3.34e-04 | Male-only specific | TBIblast < TBInonblast |
| Right caudal anterior cingulate | Cho/Cr area | TBIblast vs TBInonblast | 0.0060 | 2.04e-07 | Significant in both | TBIblast < TBInonblast |
| Right caudal anterior cingulate | Cho/Cr peak | TBIblast vs TBInonblast | Not significant | 0.0109 | Male-only specific | TBIblast < TBInonblast |
| Right paracentral region | Cho/Cr area | TBIblast vs TBInonblast | 0.0310 | 4.30e-05 | Significant in both | TBIblast < TBInonblast |
| Right posterior cingulate | Cho/Cr area | TBIblast vs TBInonblast | 0.1766 | 9.31e-05 | Male-only specific | TBIblast < TBInonblast |
| Right precuneus | Cho/Cr area | TBIblast vs TBInonblast | Not significant | 0.0031 | Male-only specific | TBIblast < TBInonblast |
| Right superior frontal cortex | Cho/Cr area | TBIblast vs TBInonblast | 0.0896 | 2.12e-04 | Male-only specific | TBIblast < TBInonblast |
| Right superior parietal | Cho/Cr area | TBIblast vs TBInonblast | Not significant | 0.0016 | Male-only specific | TBIblast < TBInonblast |

**Table S2. Sensitivity-analysis summary of TBIblast vs TBInonblast.** Two finding(s) were significant in both analyses, 0 only in the full cohort, and 14 only in the male-only analysis.

## TBIblast vs nonTBInonblast

| Brain region | MRSI measure | Group contrast | Primary Bonferroni p | Male-only Bonferroni p | Finding | Direction |
|---|---|---|---|---|---|---|
| Left cerebral white matter | Cho/Cr area | TBIblast vs nonTBInonblast | 4.72e-04 | 6.86e-05 | Significant in both | TBIblast < nonTBInonblast |
| Right cerebral white matter | Cho/Cr area | TBIblast vs nonTBInonblast | 2.50e-05 | 1.25e-05 | Significant in both | TBIblast < nonTBInonblast |
| Left caudal anterior cingulate | Cho/Cr area | TBIblast vs nonTBInonblast | 0.0399 | 0.0027 | Significant in both | TBIblast < nonTBInonblast |
| Left paracentral region | Cho/Cr area | TBIblast vs nonTBInonblast | 0.0020 | 0.0068 | Significant in both | TBIblast < nonTBInonblast |
| Left posterior cingulate | Cho/Cr area | TBIblast vs nonTBInonblast | 3.36e-04 | 3.21e-04 | Significant in both | TBIblast < nonTBInonblast |
| Left precentral cortex | Cho/Cr area | TBIblast vs nonTBInonblast | 0.0044 | 0.0112 | Significant in both | TBIblast < nonTBInonblast |
| Left precuneus | Cho/Cr area | TBIblast vs nonTBInonblast | 0.0019 | 0.0012 | Significant in both | TBIblast < nonTBInonblast |
| Left superior frontal cortex | Cho/Cr area | TBIblast vs nonTBInonblast | 2.73e-06 | 1.97e-06 | Significant in both | TBIblast < nonTBInonblast |
| Left superior frontal cortex | Cho/Cr peak | TBIblast vs nonTBInonblast | 0.0379 | Not significant | Primary analysis only | TBIblast < nonTBInonblast |
| Right caudal anterior cingulate | Cho/Cr area | TBIblast vs nonTBInonblast | 0.0023 | 4.40e-04 | Significant in both | TBIblast < nonTBInonblast |
| Right caudal middle frontal | Cho/Cr area | TBIblast vs nonTBInonblast | 0.0297 | 0.3185 | Primary analysis only | TBIblast < nonTBInonblast |
| Right paracentral region | Cho/Cr area | TBIblast vs nonTBInonblast | 0.0104 | 0.0102 | Significant in both | TBIblast < nonTBInonblast |
| Right postcentral cortex | Cho/Cr area | TBIblast vs nonTBInonblast | 2.59e-04 | 4.09e-05 | Significant in both | TBIblast < nonTBInonblast |
| Right posterior cingulate | Cho/Cr area | TBIblast vs nonTBInonblast | 0.0031 | 0.0014 | Significant in both | TBIblast < nonTBInonblast |
| Right precentral cortex | Cho/Cr area | TBIblast vs nonTBInonblast | 0.0410 | 0.0815 | Primary analysis only | TBIblast < nonTBInonblast |
| Right precuneus | Cho/Cr area | TBIblast vs nonTBInonblast | 8.52e-05 | 1.35e-05 | Significant in both | TBIblast < nonTBInonblast |
| Right superior frontal cortex | Cho/Cr area | TBIblast vs nonTBInonblast | 0.0010 | 4.97e-04 | Significant in both | TBIblast < nonTBInonblast |
| Right superior parietal | Cho/Cr area | TBIblast vs nonTBInonblast | 0.0043 | 0.0092 | Significant in both | TBIblast < nonTBInonblast |

**Table S3. TBIblast vs nonTBInonblast:** Fifteen (15 )finding(s) were significant in both analyses, 3 only in the full cohort, and 0 only in the male-only analysis.

## TBInonblast vs nonTBInonblast

| Brain region | MRSI measure | Group contrast | Primary Bonferroni p | Male-only Bonferroni p | Finding | Direction |
|---|---|---|---|---|---|---|
| Corpus callosum (central) | Cho/Cr peak | TBInonblast vs nonTBInonblast | 7.37e-04 | 0.0282 | Significant in both | TBInonblast > nonTBInonblast |
| Corpus callosum (central) | NAA/Cr area | TBInonblast vs nonTBInonblast | 0.0208 | Not significant | Primary analysis only | TBInonblast > nonTBInonblast |
| Corpus callosum (central) | NAA/Cr peak | TBInonblast vs nonTBInonblast | 0.0087 | Not significant | Primary analysis only | TBInonblast > nonTBInonblast |
| Right postcentral cortex | Cho/Cr area | TBInonblast vs nonTBInonblast | 0.0127 | 0.0065 | Significant in both | TBInonblast < nonTBInonblast |
| Right postcentral cortex | Cho/Cr peak | TBInonblast vs nonTBInonblast | Not significant | 0.0277 | Male-only specific | TBInonblast < nonTBInonblast |

**Table S4. nonTBIblast vs nonTBInonblast:** 3 finding(s) were significant in both analyses, 1 only in the full cohort, and 0 only in the male-only analysis.

## nonTBIblast vs nonTBInonblast

| Brain region | MRSI measure | Group contrast | Primary Bonferroni p | Male-only Bonferroni p | Finding | Direction |
|---|---|---|---|---|---|---|
| Corpus callosum (central) | Cho/Cr peak | nonTBIblast vs nonTBInonblast | 0.0341 | Not significant | Primary analysis only | nonTBIblast > nonTBInonblast |
| Corpus callosum (central) | NAA/Cr area | nonTBIblast vs nonTBInonblast | 0.0097 | 0.0179 | Significant in both | nonTBIblast > nonTBInonblast |
| Corpus callosum (central) | NAA/Cr peak | nonTBIblast vs nonTBInonblast | 0.0012 | 0.0026 | Significant in both | nonTBIblast > nonTBInonblast |
| Right postcentral cortex | Cho/Cr area | nonTBIblast vs nonTBInonblast | 0.0280 | 0.0096 | Significant in both | nonTBIblast < nonTBInonblast |

**Table S5. nonTBIblast vs nonTBInonblast:** 3 finding(s) were significant in both analyses, 1 only in the full cohort, and 0 only in the male-only analysis.

## TBIblast vs nonTBIblast

| Brain region | MRSI measure | Group contrast | Primary Bonferroni p | Male-only Bonferroni p | Finding | Direction |
|---|---|---|---|---|---|---|
| Corpus callosum (central) | NAA/Cr peak | TBIblast vs nonTBIblast | 0.0326 | 0.0315 | Significant in both | TBIblast < nonTBIblast |

**Table S6. TBIblast vs nonTBIblast:** 1 finding(s) were significant in both analyses, 0 only in the full cohort, and 0 only in the male-only analysis.

**Multiple-blast TBI vs non-TBI control**

| Brain Region | Primary Analysis (Bonferroni) | Male-Only Analysis (Bonferroni) | Overlap / Finding Category | Direction of Difference |
|---|---|---|---|---|
| Right cerebral white matter | 0.000028 | 0.000064 | Overlapping Region | Lower in Multiple-Blast TBI |
| Left superior frontal cortex | 0.000053 | 0.000060 | Overlapping Region | Lower in Multiple-Blast TBI |
| Left cerebral white matter | 0.000064 | 0.000030 | Overlapping Region | Lower in Multiple-Blast TBI |
| Right precuneus | 0.000083 | 0.000075 | Overlapping Region | Lower in Multiple-Blast TBI |
| Left posterior cingulate | 0.000133 | 0.000333 | Overlapping Region | Lower in Multiple-Blast TBI |
| Left paracentral | 0.001162 | 0.008416 | Overlapping Region | Lower in Multiple-Blast TBI |
| Left precuneus | 0.001467 | 0.004412 | Overlapping Region | Lower in Multiple-Blast TBI |
| Left precentral | 0.002042 | 0.002931 | Overlapping Region | Lower in Multiple-Blast TBI |
| Right posterior cingulate | 0.002464 | 0.004203 | Overlapping Region | Lower in Multiple-Blast TBI |
| Right caudal anterior cingulate | 0.004956 | 0.004531 | Overlapping Region | Lower in Multiple-Blast TBI |
| Right paracentral | 0.007046 | 0.016091 | Overlapping Region | Lower in Multiple-Blast TBI |
| Right superior frontal cortex | 0.010291 | 0.016185 | Overlapping Region | Lower in Multiple-Blast TBI |
| Right postcentral | 0.011192 | 0.007157 | Overlapping Region | Lower in Multiple-Blast TBI |
| Left caudal anterior cingulate | 0.028069 | 0.006035 | Overlapping Region | Lower in Multiple-Blast TBI |
| Left caudal middle frontal | 0.035052 | 0.036675 | Overlapping Region | Lower in Multiple-Blast TBI |
| Right superior parietal | 0.010108 | 0.065254 | Primary Analysis Only | Lower in Multiple-Blast TBI |
| Right caudal middle frontal | 0.025202 | 0.404072 | Primary Analysis Only | Lower in Multiple-Blast TBI |
| Left isthmus cingulate | 0.051932 | 0.027098 | Male-Only Specific | Lower in Multiple-Blast TBI |

**Table S7. Sensitivity analysis. Multiple-blast TBI vs non-TBI control.** The table compares Bonferroni-adjusted results for the TBI-versus-non-TBI contrast in the full cohort with the corresponding male-only analysis. Rows are restricted to source spreadsheet entries labeled “nonTBI.” When a region appeared more than once in a source spreadsheet, the smallest Bonferroni-adjusted p-value among the non-TBI entries was used for the regional summary. Fifteen (15) regions were significant in both analyses, 2 were significant only in the primary analysis, and 1 was significant only in the male-only analysis. All summarized significant effects were in the direction of lower Cho/Cr area in Multiple-Blast TBI relative to non-TBI controls.
